# Experimental Assessment of Hemp Shiv and Green Adhesives to Produce a Biocomposite Material

**DOI:** 10.3390/ma17163900

**Published:** 2024-08-06

**Authors:** Borja Martínez, Virginia Mendizabal, Ernest Bernat-Masó, Lluís Gil

**Affiliations:** Department of Strength of Materials and Structures in Engineering, Polytechnic University of Catalonia, 08222 Terrassa, Spain; borja.martinez@upc.edu (B.M.); virginia.dolores.mendizabal@upc.edu (V.M.); ernest.bernat@upc.edu (E.B.-M.)

**Keywords:** hemp shiv, biocomposite material, mechanical performance, sustainability

## Abstract

This study investigated the utilization of innovative green composites made from hemp shiv, a waste by-product of hemp cultivation, with the aim of promoting sustainability within the construction industry. The manufacturing method involved the application of pressure in a mold to create the samples. These materials were produced using an environmentally friendly binder consisting of colophony, arabic gum, and corn starch. Moreover, white glue and bioepoxy were also used to compare with the green resins. Three different binder compositions were used for the specimens. The samples underwent mechanical testing through tensile and bending assessments, and their performance was compared to that of non-green binders to validate the effectiveness of the manufacturing processes. The study revealed that decreasing the moisture content during the curing process was crucial for improving the mechanical properties. The best results were achieved when using arabic gum as a binder, yielding a tensile strength of 2.16 MPa and a bending strength of 5.25 MPa, with a composition of 62.5% hemp shiv and a manufacturing process involving a pressure of 5 MPa.

## 1. Introduction

Over recent decades, the negative effects of climate change have become a significant problem for our society. In this context, the scientific community must focus on researching new eco-friendly, sustainable, and renewable materials [1]. The scientific community is now actively aiming to develop novel materials that align with contemporary energy and environmental criteria. Hence, the imperative to discover new bio-based materials that are both energy-efficient and cost-effective, while boasting reduced carbon footprints [2]. Numerous plant-based materials have been explored, revealing hemp as one of the most promising options. With its resilience to drought and minimal need for fertilization, hemp can be cultivated effectively across numerous countries [3]. Moreover, hemp is a versatile crop with a wide range of applications, spanning multiple industries.

Hemp shiv is often regarded as a low-value by-product of the hemp crop, primarily because there are insufficient applications to fully utilize the material [4]. It constitutes more than 50% of the total weight of the crop [5]. Recent publications have suggested that lignin-based resin, bio-epoxy resin [6], and recyclable cardboard fiber hold promise as potential binding materials for creating a novel bio-composite material using hemp shiv [7]. Due to its nature, materials made with wood chips can be extrapolated to provide an initial solution for hemp shiv-based materials. The elastic modulus of hemp shiv (10–16 GPa) varies depending on its position within the stem of the plant, determined by its height [8]. However, the specific region with the highest elastic modulus differs across hemp species. These variations in elastic modulus are associated with changes in the size of the cell wall along the stem [9], resulting in a a variability in results depending on the properties of the local product.

Different applications are being studied to use hemp shiv, taking advantage of its low price, such as using it as insulation in buildings [10,11]. Hempcrete is manufacture by adding lime and water to the hemp shiv in order to form non-structural blocks [12]. A sustainable substitute for traditional walls, a study of the acoustic absorption properties of lime and hemp shiv walls was carried out, obtaining an average of between 40 and 50% acoustic absorption [13]. The main advantages of hempcrete is the insulating properties provide by the hemp shiv, while the lime binder provides a protection against moisture, fungi, and fire [14]. With the same approach, adding hemp particles to mortar as aggregates can reduce its density and increase the insulating properties, and also the material will increase the capability for CO_2_ storage; nevertheless, the mechanical properties decrease (maximum stress is reduced up to 30% when adding 8% hemp) [15,16,17]. Although cementitious matrices offer the benefits of affordability and adaptability, their use can result in chemical damage to hemp shiv [18].

Hemp shiv particles are also used as a raw material for materials made up of wood particles. In this way, the manufacturing process is to mix it with a binder to fabricate a material similar to chipboard. The manufacture of this type of material consists of mixing the shiv with the binder material and applying pressure and temperature in a mold, where the adhesive is cured and the material obtains the shape of the mold. The most commonly used binders are currently based on formaldehyde, because of its mechanical properties, dynamic properties, abrasion resistance, and affordability [19]. Nevertheless, due to the fact that it is a toxic material in large quantities, its use has been decreasing. An intermediate solution is the use of two formaldehyde-based adhesives by partially substituting them for lignocellulose-based materials (wheat straw, and pine and poplar particles) obtaining better results with PDMI (Polymeric Diphenylmethane Diisocyanate), by increasing the percentage of binding material [20,21]. PDMI showed better binding properties than UF (urea formaldehyde) when curing at a temperature of 180 °C and applying pressure for 3 min [22], and these are the usual values in the industry. The process is also the same with vegetable agglomerate, taking into account the curing temperature for each vegetable binder [23,24]; nevertheless, the curing time is higher for vegetable resins. In this type of manufacturing, the structure and size of the particles is also an important factor in the final properties of the material. If the particle size is very large, air gaps will be produced in the material, as all the chips cannot be compacted together because the manufacture process do not use a vacuum to prevent air gaps. However, this can be solved by including saw and wood dusts that occupy these holes together with the resin, thus improving the mechanical properties of the material [25].

Another alternative is to completely eliminate formaldehyde-based resins by using natural ones. Studies are being carried out to obtain vegetable resins that can achieve the regulatory requirements for different applications, such as lignin-based wood adhesives [26,27], obtaining materials with good thermal properties, or vegetable proteins such as camellia protein, which is also a residue in biodiesel production [28]. However, the main problem is the resistance to fire. This problem can be solved by adding a fire resistance coating. There have also been studies to develop a sustainable, high-performance, and flame-retardant wood coating based on a curing agent of ammonium hydrogen phytate (AHP) [29,30]. Starch is also a good bio-based binder for wood particles; for example, a cassava starch binder can be use to elaborate a low-density particleboard with excellent performance [31]. The fungal resistance is low; however, citric acid can be added to improve the fungal degradation by 10% [32].

This study focused on research solutions of green materials based on hemp shiv. Therefore, specimens made with hemp shiv and different green binders were tested though compression and bending. Moreover, several fabrications methods were employed in order to study the characteristics that improved the mechanical properties of the material. The study aimed to improve on existing knowledge of hemp-based materials by using two adhesives (colophony and arabic gum) that are not found in the literature and comparing them with other adhesives that are being studied (starch, bioepoxy, and white glue).

## 2. Materials

### 2.1. Hemp Shiv

**Figure 1:**
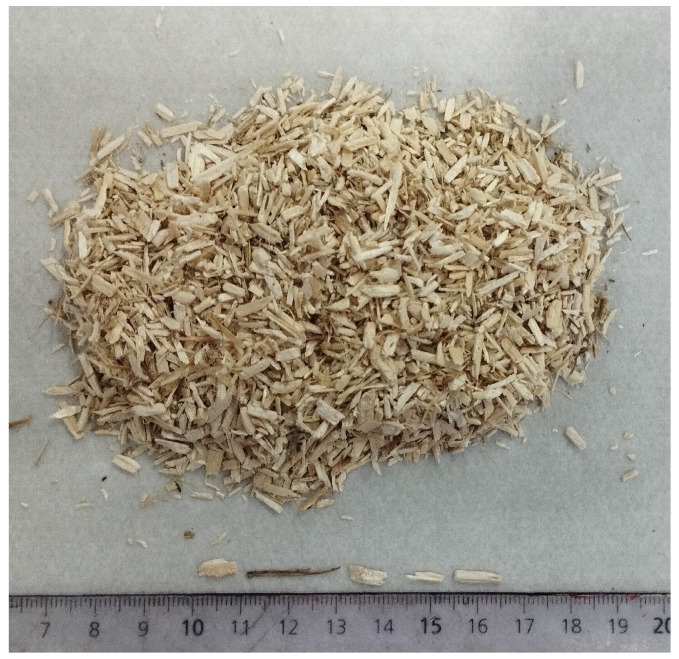
Hemp shiv size distribution.

Hemp shiv, Figure 1, refers to the woody portion of the plant’s trunk. The hemp shiv used in this research was provided by Planteles Lloveras, a local harvesting company. The hemp shiv particles exhibited a size distribution ranging from 5 to 20 mm in length. In certain samples, specifically the smaller particles were gather, measuring between 5 and 10 mm in length.

#### 2.1.1. Colophony

Colophony, an abundant and cost-effective natural resin derived from pine trees, is a renewable and biodegradable resource with low molecular weight. Colophony was dissolved in acetone in 2 proportions and 2:1 and 1:1 ratios at 50 °C to produce the binder.

#### 2.1.2. Arabic Gum

Arabic gum, or acacia gum, is an exudate produced by acacia trees in sub-Saharan countries, which operates as a natural wound plaster, thus shielding trees against insects, molds, and drought. It is a highly water soluble material [33]. Arabic gum was dissolved in water in 2 proportions and 2:1 and 3:2 ratios at 90 °C to produce the binder.

#### 2.1.3. Corn Starch

Starch is a carbohydrate. When mixed with hot water, it creates a wheat-like dough, frequently used as a thickening agent, stiffener, or adhesive. The adhesive was produced following proportions outlined in the literature, with a ratio of 100 g of water for every 18 g of starch at 65 °C, and the mixture was subjected to temperature and agitation [34].

#### 2.1.4. Bioepoxy

ONE super sap is a epoxy resin made with 30% green materials, in order to corroborate the mechanical properties of an intermediate solution. The resin is a general purpose laminating resin with high bio-based content for composite laminating, coating, and adhesive applications.

#### 2.1.5. White Glue

White glue HM-425 served as a reference material, since it ranks among the most frequently employed inorganic adhesives for wood-based products. It is a water-based adhesive, rich in polyvinyl acetate (PVA).

## 3. Methodology

### 3.1. Fabrication Method

**Figure 2:**
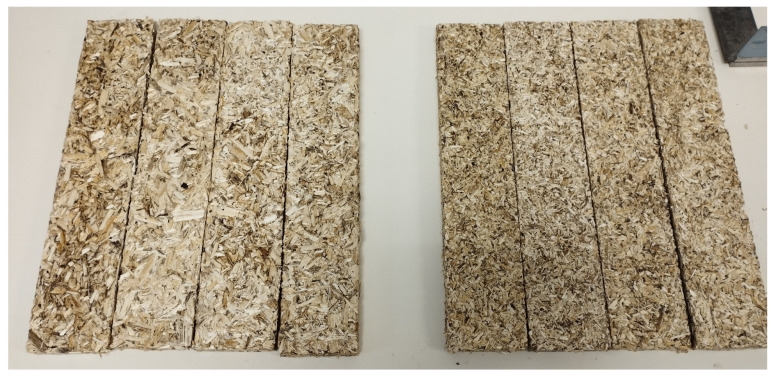
Specimens made with arabic gum.

To conduct the study, composite specimens were fabricated using different resin selections and three different compositions (10 g of hemp shiv with 2/4/6 g of binder) to verify the stability of the resulting material, Figure 2. These ratios were used to verify the mechanical properties with different amounts of binder, taking into account that commercial materials use a binder ratio of 5–10%. Nevertheless, samples with less than 15% of the proposed green materials were unstable, which was the reason for starting with a 10-2 ratio. Subsequently, mechanical characterization tests were performed to determine the best proportions and binders.

To make the specimens, two steel molds were used to contain the hemp shiv mixed with the previously prepared binder, and pressure was applied from below to obtain the correct dimensions. In the case of vegetable resins, temperature does not reduce the curing time. To make an initial approximation of the best selection, it was decided to apply 5 MPa pressure within the molds for 5 min instead of applying pressure during the entire curing time, completing the curing process outside the molds at ambient conditions.

Favorable results were obtained with the five resins tested, allowing subsequent mechanical tests to be carried out. However, it was observed that curing outside the mold at ambient temperature caused the specimens to increase in size during curing. Consequently, the properties obtained would be less to those achieved when pressure is applied throughout the entire curing process. In addition, a highly viscous binder, such as white glue, presented challenges during the mixing process of the two materials, especially when used in small quantities, leading to a non-homogeneous mixture.

Notably, the specimens made with arabic gum, which utilizes water, exhibited the most significant changes in dimensions. Specimens with higher proportions of binder tended to break due to the significant increase in dimensions. This phenomenon occurred because the hemp absorbed water from the resin, leading to volume expansion.

Due to the similar behavior observed in the samples after the mixing performance test, an optimization study of the fabrication method was conducted using the most promising resin (arabic gum). Subsequently, this optimization process was extended to the other resins that were utilized. The primary emphasis of the optimization for vegetable-based resins was placed on refining the manufacturing process and managing humidity levels during the curing stage, as these were the primary challenges encountered during the initial fabrication.

The optimization process consisted in solving the problems presented in the initial methodology, involving the water absorption problem and the curing time. Therefore, five modifications were proposed:

- Concentrate the resin. In order to reduce the moisture during the curing phase.
- Introduce absorption paper and a wood mold to increase the curing time up to 1 week. The absorption paper reduced the moisture inside the mold during the curing time and the wooden mold was more breathable than the steel mold, which helped reduce the interior humidity, allowing the sample to spend more time curing in the mold.
- Increase the pressure manufacturing time. To reduce the volume expansion.
- Reduce the hemp shiv size. To increase the mechanical strength.
- Curing time in oven at 120 °C for 1 h. To evaporate the water of the resin while the sample was in the mold.

### 3.2. Specimens

To introduce the specimens produced, they were labeled according the composition, binder, and manufacture method, Table 1. In that way, the samples were labeled with one letter from each list according to the methodology used to manufacture them.

### 3.3. Test Method

#### 3.3.1. Tensile Test

**Figure 3:**
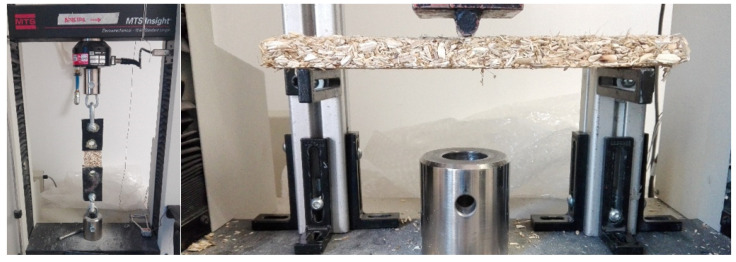
Test configuration: tensile (**left**) and bending (**right**).

The test procedure followed the regulations outlined in EN 319 [35]. Prismatic specimens with dimensions of 50 × 50 × 5 mm were used. A tensile test was conducted using an electromechanical universal testing system of 10 kN, with a constant test speed of 5 mm/min. The test setup included a self-aligning ball and socket joint component positioned within the testing machine and connected to a wooden block to exert force on the sample, as shown in Figure 3. The sample was affixed to the wooden block using white glue to ensure that the failure occurred within the sample itself and not at the bonding interface.

#### 3.3.2. Bending Test

A 3-point bending test configuration was employed, Figure 3, with a distance of 200 mm between the supports. The test procedure followed the regulations outlined in EN 310 [36]. Prismatic specimens with dimensions of 250 × 50 × 10 mm were tested. A bending test was conducted using an electromechanical universal testing system of 10 kN, with a constant test speed of 5 mm/min. The formulas described below correspond to the standard used, where a linear stress distribution was assumed. It is important to note that this assumption does not hold for the proposed material. However, the formulation was retained to facilitate comparisons between the test specimens and commercially available results.

## 4. Results and Discussion

The outcomes of the preliminary specimens are presented in Table 2. However, it should be noted that certain samples became unstable and broke on handling before testing, leading to values in the table without a coefficient of variation. Of particular significance is the case of starch resin, where all the samples prepared proved to be unstable due to the high moisture content of the resin, causing the samples to swell during the curing process, so it was removed from the table.

The primary objective of the initial results was to establish the appropriate blending protocol and validate the specimen fabrication. For non-vegetable resins, an increase in resin quantity corresponds to improved tensile and bending properties. For the white glue, the 2–10 composition (LW-2-I) exhibited a low binder content, producing a good mix.

However, this pattern did not hold true for the vegetable resins. In the case of the 6–10 composition with arabic gum and colophony (LA-6-I & LC-6-I), the tensile samples were unstable. This discrepancy was attributed to residual moisture in these resins, which led to an enlargement of hemp shiv and an expansion of the specimen dimensions during air curing.

Both the vegetable and synthetic resin results obtained fell significantly below the benchmarks set by commercial materials (tensile strength: 0.4 MPa & bending strength: 11 MPa [37]), underscoring the need for production enhancements. Subsequently, the focus shifted toward refining the conducted processes to minimize specimen numbers. These interim steps exclusively involved vegetable resins. Once the final process had been selected, specimens of all types could be produced to facilitate comparative analysis.

Based on the outcomes presented, modifications in the manufacture process were proposed to improve the quality of the samples. In terms of composition, the mechanical strength increased with the amount of applied resin. In the following samples, the compositions were reduced to 10-2/6.

The first suggestion was to concentrate the resins, for the purpose of minimizing moisture within the mold. Initially, the ratio was 2 g of water per 1 g of arabic gum; however, in these instances, a 3:2 ratio was adopted. This proportion was applied to two different cases, 2–10 and 6–10 (HA-2-I & HA-6-I). In both cases, the dimension expansion of the specimens decreased during curing. This effect was more pronounced in the 6–10 case, where a greater reduction in water was achieved, resulting in improved stability.

By using concentrated resin with a lower amount of water, the arabic gum also achieved better mechanical resistance with a higher amount of resin, Table 3. Conversely, in cases of lower resin content, the diminished water usage (and subsequently lower reduction in overall water quantity), as well as the challenge in blending the two components with a smaller amount, produced a reduction in the mechanical properties compared to the non-concentrate case. Based on these findings, it was decided to utilize concentrated resin exclusively in the scenario of the 6–10 composition. This adaptation resulted in a reduction of 30 g of water within the bending specimens.

A further recommendation was to prolong the duration of pressure application within the steel mold for 5 h at 5 MPa, which was then left to air dry for 1 week (LA-2-P). This approach aimed to mitigate sample expansion by allowing more time for resin curing, resulting in higher strength upon specimen demolding. Another approach consisted of 5 min pressure application in the steel mold and then the mold was introduced into a 120 °C oven for 1 h (HA-6-O). Similarly to the previous modification, this method aimed to expedite sample drying, thereby reducing the curing time that the specimen remained in the mold. Samples were also fabricated using smaller-sized hemp shivs (HA-6-T). To obtain finer hemp shivs, the sample was filtered through a sieve, resulting in a median particle size of 5 mm compared to the initial 10 mm medium length. Table 4 shows the results of the different fabrication methods proposed.

Unfortunately, the achieved outcomes were not favorable, mainly because the 5 h duration within the mold proved inadequate for the complete resin curing process. After removing the specimen from the mold, it was observed that the humidity inside the mold was substantial. Moreover, during the week of curing outside the mold at ambient conditions, the dimensions increased significantly. This phenomenon occurred because the binder began to cure inside the mold, causing the hemp to absorb the remaining water, as the mold did not allow moisture to escape. The modification aiming to improve the curing process through elevated temperatures failed to enhance specimen outcomes, as this approach led to the development of internal cracks caused by the swift removal of water. The filtration of hemp shiv in order to use smaller particles did not contribute to improved results. Using smaller hemp particles can enhance the mechanical properties in this composite material [38]; however, the hemp shiv used was already within the described small particle range. In that case, further reducing the particle length produced a reduction in the maximum strength of the sample. Consequently, these attempted modifications were disregarded. The hemp shivs were soaked for 24 h to prevent further moisture absorption from the binder and to prevent volume changes. Nevertheless, utilizing damp hemp shivs hindered the effective curing of the arabic gum, leading to instability of the specimens.

**Figure 4:**
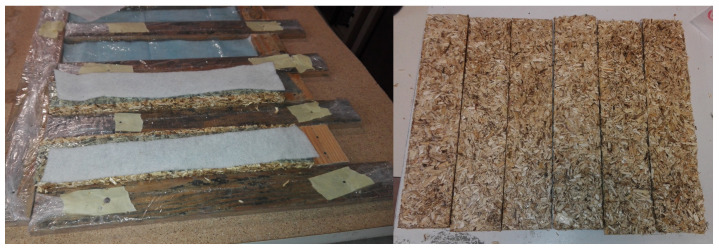
Wooden mold with bleeding and absorbent paper (**left**) and obtained specimens (**right**).

Based on these results, it was proposed to keep a sample in the mold for 1 week applying pressure in order to maintain the shape (HA-6-W), and to use bleeding paper and absorbent paper similar to those used in composite material fabrication with infusion methods, to reduce the quantity of water that was absorbed by the hemp shiv, Figure 4. The wood mold and absorbent paper reduced the moisture absorbed by the hemp, and since the specimen remained in the mold during curing, it maintained its dimensions. With these two processes, the dimensions improved from 15 mm to 10–11 mm, increasing the compactness of the samples, resulting in improved properties, as shown in Table 5. These processes were extrapolated to the other initially considered resins.

Furthermore, for the arabic gum and colophony composites, a two-step curing process was proposed using the wooden mold. This was because colophony, which utilized acetone, did not exhibit as many moisture-related issues. However, it is important to note that the optimal curing time varied for each resin, due to their distinct curing mechanisms, as indicated in Table 5. For arabic gum, water absorption by the wood and paper components creates a high-moisture environment over an extended period, which can decrease the strength of arabic gum, and the hemp shiv may also absorb moisture, reducing its mechanical properties. In contrast, colophony undergoes elimination upon contact with air. In the mold, the evaporation process is slower, resulting in a more resilient sample with one week of curing time. In this scenario, the most favorable outcomes were achieved, with values approaching those of commercial materials (tensile strength: 0.4 MPa & bending strength: 11 MPa [37]). Consequently, this modification was implemented for the other binders as well.

In the initial stages, corn starch was utilized as a binding agent. However, the material exhibited instability due to the significant amount of water required for the binder. Through refining the manufacturing process, which involved employing a wooden mold with a drainage system and allowing for one week of drying time with absorbent paper, the mixture became sufficiently stable for mechanical testing (LS-0.5-D & LS-1-D). The results obtained from using food-grade starch, coupled with this optimized manufacturing approach, are detailed in Table 5. Nevertheless, these outcomes fell short of those achieved with the two vegetable resins applied. In the case of 1–10, the samples remained unstable due to the high water content in the mixture, even with the improved fabrication method.

**Figure 5:**
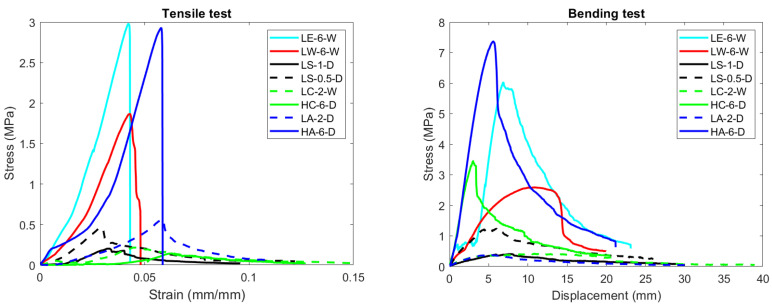
Results of tensile (**left**) and bending (**right**) tests for the best cases.

In Table 6 and Figure 5, a comparison is presented between all the resins employed in the study and the top-performing scenarios. The fabrication process involved applying pressure for 5 min, followed by placing the sample in a wooden mold with absorbent paper for curing. For white glue, bioepoxy, and colophony, the curing period was 1 week, while for arabic gum and corn starch, it was 1 day. For the case of colophony and arabic gum, the concentrated resin was used. Analyzing these findings reveals that, when compared to commercial benchmarks, the attained tensile strength improved the best instances by a factor of up to 5. Nonetheless, the bending strength remained within the range of 50% of that seen in commercial boards (tensile strength: 0.4 MPa & bending strength: 11 MPa [37]) for the most successful cases, arabic gum 6–10 (HA-6-D). The medium density of the samples manufactured was 480–500 kg/m^3^, obtaining a lower density comparing to commercial chipboard, 550–800 kg/m^3^. The difference in density and the volume expansion presented in the sample indicated that the curing process can still be improved to obtain samples with better mechanical performance. Regarding the attained outcomes, the current material’s suitability as a particle board is limited due to its lower bending mechanical characteristics. Considering the best case scenario with arabic gum, the bending Young’s modulus was 50% lower than commercial materials (1800 MPa [37]). Additionally, the amount of binder used was higher, with a 5–10% ratio in commercial chipboards [37].

Nevertheless, with this mechanical performance, this green composite material could be used in some applications in the construction sector. The coffered ceiling is a suitable application, because its mechanical properties are suitable, with a similar density to commercial materials and moreover it also has insulating properties that could be interesting for these applications. The attained mechanical strength values met regulatory standards, and its bending strength was two times greater than EPS (150 Kpa) in the case of 2–10 with colophony (350 kPa). Colophony was chosen for its hydrophobic properties; moreover, colophony protects the cellulose, hemicellulose, and lignin from alkaline environments [39,40]. Therefore, it shields the hemp shiv from the alkaline environment created by concrete. It is necessary to protect the material during the initial week to prevent degradation of its mechanical properties. In the case of arabic gum, since it is soluble in water, the material may not be resistant enough to withstand the weight of the concrete until it is fully cured. Nevertheless, it is crucial to acknowledge that an excessively alkaline environment could also break down the vegetable binder, hinder its advantages, and produce an unstable material.

After the concrete is poured and undergoes curing, these blocks become an integral component of the structure. It is crucial to verify material compatibility and ensure that the green material remains stable for at least 7 days, allowing the concrete curing process to take place without degradation of the mechanical properties of the bio-composite or the concrete.

Table 7 presents a comparison of the compression Young’s modulus and bending strength of the hemp-based materials. The mechanical properties of the composite were significantly influenced by the binder used. It is interesting to note that introducing vegetable materials into a matrix like cement significantly reduces the mechanical properties of the material, even to the point of making it inefficient for use as a structural material. In such cases, using a material that is 100% vegetable for non-structural applications increases the options for introducing more eco-friendly materials into the construction industry.

The material developed in this study showed a good performance for non-structural applications in constructions compared to other green materials. It could be use in some applications replacing materials like chipboard or wood-based materials with similar mechanical performance or EPS. Increasing the use of renewable materials in the construction sector with these materials.

## 5. Conclusions

In this study, an experimental investigation was conducted to explore a biocomposite material based on hemp shiv. The experimental campaign included variations of the manufacture process and materials in samples tested by tensile and bending tests. The main conclusions of the study are as follows:

- The use of manufacturing processes and materials with low water content increased the mechanical performance of the composites made with hemp shiv.
  The mechanical properties were greatly influenced by moisture during the curing process, as the hemp shiv absorbed water, leading to swelling and a reduction in mechanical properties. Therefore, employing techniques to minimize moisture content in the material during curing is recommended.
- The application in coffered ceilings is feasible using colophony as binder.
  While the mechanical properties of green resins may not match chipboard, they are adequate for coffered ceiling applications, achieving a bending strength of 2.77 MPa in the HC-6-W case. Hence, colophony is recommended, due to its water and moisture resistance. For particleboard application, structural reinforcement is necessary to improve the bending strength. An option to explore is to introduce a vegetable mesh into the material.
- The application of chipboard could be suitable for hemp-shiv-based materials using arabic gum as a binder.
  Since the bending strength of the materials is lower (5,25 MPa) than commercial materials, the dimensions of the materials should be larger. It is also necessary to introduce a superficial organic coating to protect the materials against ambient water during its application. Moreover, a structural reinforcement is necessary to improve the bending strength.

Gathering all this information, it can be concluded that in some applications in the construction industry, hemp shiv could be use as a more ecological material to substitute the inorganic commercial materials. Coffered ceilings could offer a great starting point into the construction sector for hemp shiv. However, it should be noted that the use of cementitious matrices can lead to chemical damage to the hemp shiv and also to the colophony coating applied. A solution that could be studied in future research is to apply a superficial less alkaline matrix to mortar in order to reduce the alkalinity at the interface of the two materials.

## Figures and Tables

**Table 1:** Nomenclature of the specimens.

Resin	Relation Hemp-Binder	Manufacture Method
LA (Arabic gum)	0.5 (10-0.5)	I (initial method)
HA (Concentrated arabic gum)	1 (10-1)	P (Increase the pressure time)
LC (Colophony)	2 (10-2)	O (Curing 1 h in oven)
HC (Concentrated colophony)	4 (10-4)	D (1 day in wood mold)
LS (Corn starch)	6 (10-6)	W (1 week in wood mold)
LE (Bioepoxy)		T (thin particle size)
LW (White glue)		

**Table 2:** Tensile and bending test results.

Sample	Tensile Strength (kPa), C.V. (%)	Bending Young Modulus (MPa), C.V. (%)	Bending Strength (kPa), C.V. (%)
LW-2-I	45.7 (15)	-	-
LW-4-I	109.5 (40)	21.27 (-)	150.6 (-)
LW-6-I	279.5 (-)	39.70 (-)	491.9 (1)
LE-2-I	19.5 (35)	2.12 (29)	317 (45)
LE-4-I	139.7 (15)	59.16 (26)	208.9 (23)
LE-6-I	298.6 (26)	44.85 (1)	317 (36)
LA-2-I	174.1 (40)	32.74 (9)	150.7 (5)
LA-4-I	46.3 (5)	31.91 (3)	106.3 (6)
LA-6-I	-	45.97 (47)	202.6 (26)
LC-2-I	22.8 (23)	75.55 (28)	195.8 (34)
LC-4-I	33.1 (28)	136.48 (16)	486.2 (33)
LC-6-I	-	148 (12)	586.3 (4)

**Table 3:** Tensile and bending test results with concentrated arabic gum.

Sample	Tensile Strength (kPa) (C.V.)	Bending Young Modulus (MPa) (C.V.)	Bending Strength (kPa) (C.V.)
LA-2-I	174.1 (40)	32.74 (9)	150.7 (5)
HA-2-I	109.2 (9)	34.01 (3)	219.5 (12)
LA-6-I	-	45.97 (47)	202.6 (26)
HA-6-I	397.3	23.01 (8)	339.9 (5)

**Table 4:** Results of tensile and bending tests varying the manufacturing methods.

Sample	Tensile Strength (kPa), C.V. (%)	Bending Young Modulus (MPa), C.V. (%)	Bending Strength (MPa), C.V. (%)
LA-2-I	174.1 (40)	32.74 (9)	0.15 (5)
LA-2-P	10.9 (-)	12.55 (-)	0.11 (-)
HA-6-I	1802.3 (45)	222.79 (1)	0.74 (1)
HA-6-O	913 (15)	398.46 (5)	4.09 (18)
HA-6-T	327.4 (28)	133.15 (28)	30.58 (26)

**Table 5:** Results of tensile and bending tests varying the curing times in the mold with absorbent paper.

Sample	Tensile Strength (MPa), C.V. (%)	Bending Young Modulus (MPa), C.V. (%)	Bending Strength (MPa), C.V. (%)
HA-6-W	1.80 (45)	222.79 (1)	0.74 (1)
HA-6-D	2.16 (18)	747.19 (29)	5.25 (24)
HC-6-W	0.61 (13)	594.54 (40)	2.77 (34)
HC-6-D	0.26 (65)	558.28 (8)	1.33 (15)
LS-0.5-D	0.31 (56)	172.70 (24)	1.05 (19)
LS-1-D	0.03 (-)	21.31 (-)	0.12 (-)

**Table 6:** Results of tensile and bending tests for the best cases.

Sample	Tensile Strength (MPa), C.V. (%)	Bending Young Modulus (MPa), C.V. (%)	Bending Strength (MPa), C.V. (%)
LW-6-W	1.06 (49)	194.20 (22)	2.22 (23)
LE-6-W	1.73 (42)	338.73 (2)	5.15 (24)
LA-2-D	0.37 (32)	54.70 (3)	0.25 (35)
HA-6-D	2.16 (18)	747.19 (29)	5.25 (24)
LC-2-W	0.12 (63)	67.72 (9)	0.35 (30)
HC-6-D	0.61 (13)	594.54 (40)	2.77 (34)
LS-0.5-D	0.31 (56)	172.70 (24)	1.05 (19)

**Table 7:** Compression and bending performance compared with other shiv based materials.

Fiber	Matrix	Eco-Friendly Material	Compression Young Modulus (MPa)	Bending Strength (MPa)	Reference
Hemp shiv	Arabic gum	YES	–	5.25	This study
Hemp shiv	Cardboard	YES	0.67–0.96	0.43	[41]
Hemp shiv	Wheat starch	YES	0.55	0.08–0.14	[34]
Hemp shiv	Reactive vegetable protein	YES	1.1–3.0	6.83	[42]
Corn shiv	Epoxy	NO	0.11–0.29	0.13	[43]
Hemp shiv	Portlandt & MgO-cement	NO	0.4–5.5	–	[15]
Hemp shiv	Lime	NO	0.3–0.5	0.14	[12]

## Data Availability

The original contributions presented in the study are included in the article, further inquiries can be directed to the corresponding author.
